# Scale and shape issues in focused cluster power for count data

**DOI:** 10.1186/1476-072X-4-8

**Published:** 2005-03-31

**Authors:** Robin C Puett, Andrew B Lawson, Allan B Clark, Tim E Aldrich, Dwayne E Porter, Charles E Feigley, James R Hebert

**Affiliations:** 1 Department of Epidemiology and Biostatistics, Arnold School of Public Health, University of South Carolina, Columbia, SC, USA; 2 Department of Environmental Health Sciences, Arnold School of Public Health, University of South Carolina, Columbia, SC, USA; 3 School of Medicine, Health Policy and Practice, University of East Anglia, UK; 4 South Carolina Statewide Cancer Prevention & Control Program, Hollings Cancer Center, Medical University of South Carolina, Charleston, SC, USA

## Abstract

**Background:**

Interest in the development of statistical methods for disease cluster detection has experienced rapid growth in recent years. Evaluations of statistical power provide important information for the selection of an appropriate statistical method in environmentally-related disease cluster investigations. Published power evaluations have not yet addressed the use of models for focused cluster detection and have not fully investigated the issues of disease cluster scale and shape. As meteorological and other factors can impact the dispersion of environmental toxicants, it follows that environmental exposures and associated diseases can be dispersed in a variety of spatial patterns. This study simulates disease clusters in a variety of shapes and scales around a centrally located single pollution source. We evaluate the power of a range of focused cluster tests and generalized linear models to detect these various cluster shapes and scales for count data.

**Results:**

In general, the power of hypothesis tests and models to detect focused clusters improved when the test or model included parameters specific to the shape of cluster being examined (i.e. inclusion of a function for direction improved power of models to detect clustering with an angular effect). However, power to detect clusters where the risk peaked and then declined was limited.

**Conclusion:**

Findings from this investigation show sizeable changes in power according to the scale and shape of the cluster and the test or model applied. These findings demonstrate the importance of selecting a test or model with functions appropriate to detect the spatial pattern of the disease cluster.

## Background

Over the past several years, there has been an increased interest in the detection of focused clusters of disease, or disease clusters associated with a known pollution source [1]. Along with this interest, the development and use of various cluster detection methods have grown. Wartenberg and Greenberg [2] emphasized the importance of statistical power as a criterion in the selection of appropriate method for cluster investigations. A limited number of power evaluations in the literature have examined the issues of scale or shape with regard to focused disease cluster detection. As part of a more extensive evaluation, Sun [3] examined the power of Stone's [4] and Tango's Tests [5] to detect clusters of varying size. Waller [6] described two shapes of clusters, hot spot and clinal [2], and evaluated the power of Stone's and Besag and Newell's Tests to detect these types of clusters at varying levels of aggregation. Additional focused cluster shapes also have been proposed, such as a cluster of angular shape which could result from the effect of a dominant wind dispersing environmental contaminants [7]. However, this cluster shape has not been included in previous power evaluations. Additionally, information is lacking regarding the power of generalized linear models to detect focused clusters of varying scale and shape. This investigation evaluates the power of a number of focused cluster tests and generalized linear models to detect clusters of varying scale and shape. Count data are simulated for three total numbers of events (N = 200, 500 and 1000) to represent clustering around a single, centrally located pollution source in spatial patterns consistent with pollution dispersion principles.

## Results

### Focused cluster test results

#### Data simulated with distance decline (DD) in relative risk

**Figure 1:**
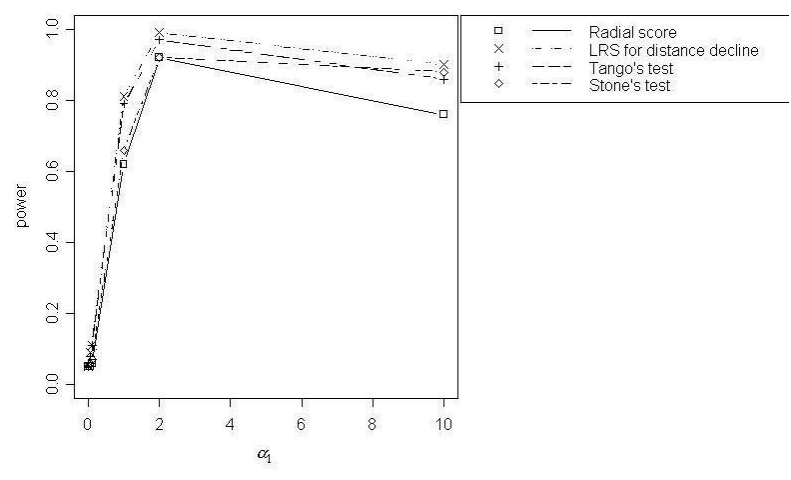
Power curves for DD with *N *= 1000

The LRS Test for Distance Decline, Tango's Focused Test, Stone's Test and the Radial Score Test demonstrated the best power curves for the range of total simulated events (*N *= 200, 500, 1000). Stone's Test showed slightly less power than the other best-performing tests; and for *N *= 500 and *N *= 1000 events the Radial Score Test performed with slightly lower power than Tango's Focused Test and the Radial Score Test. Power of 100% was reached only by Tango's Test (*τ *= 1 and 5) and the LRS Test for Distance Decline in the *N *= 1000 simulation at *α*_1 _= 2 (Figure 1). For all total event scenarios, power curves for the four best-performing tests were similar, with low power at lower values of *α*_1 _(< 2), peaking at *α*_1 _= 2 and decreasing slightly at *α*_1 _= 10. For *N *= 500 and *N *= 1000, Cuzick and Edwards' Test reached modest power ranging from 30% to 50% at the highest values for *α*_1_. As opposed to the focused cluster tests that showed the best power curves, the power for Cuzick and Edwards' Test continued to increase up to the highest value of *α*_1 _(*α*_1 _= 10) rather than declining slightly. All other focused cluster tests that were examined showed less power than those previously described.

#### Data simulated with peaked distance decline (PDD) in relative risk

**Figure 2:**
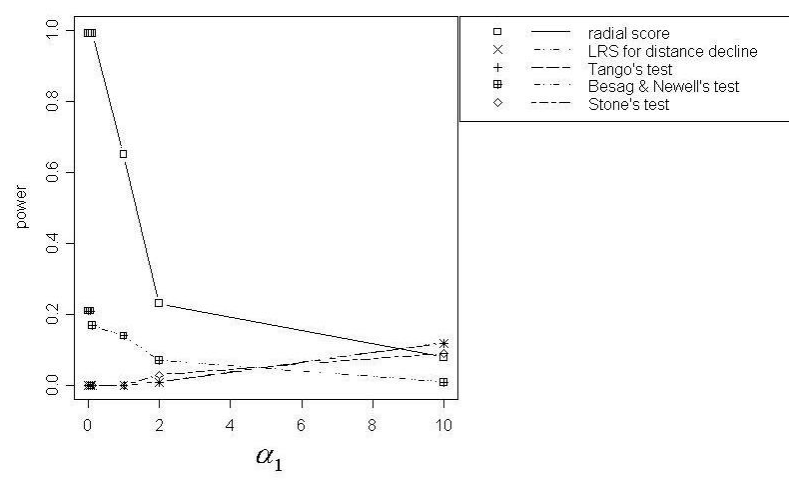
Power curves for PDD, *α*_2 _= 1, with *N *= 1000

The LRS Test for Distance Decline, Tango's Test, the Radial Score Test, Besag and Newell's Test, Stone's Test, and Cuzick and Edwards' Test showed the best power curves for PDD clusters. However only modest power was achieved (50%) for *N *= 200 by the focused cluster tests with the best power curves: LRS Test for Distance Decline, Tango's Test, and the Radial Score Test. Power increased with increasing events. Eighty percent power was achieved for *N *= 500, and 100% power was achieved for *N *= 1000 (Figure 2). As expected, power curves were similar to those for DD at the lowest values of *α*_2 _(*α*_2 _< 0.5), with power increasing as *α*_1 _increased until a slight drop at the highest *α*_1 _value (*α*_1 _= 10). However as *α*_2 _increased, the power curves changed. For *N *= 200, power curves for *α*_2 _= 0.5 and *α*_2 _= 1 were similar and showed the Besag and Newell's Test reached the highest power of any focused cluster test at lower values of *α*_1_. Power for this test declined as *α*_1 _increased; whereas the power increased for Tango's Test, Stone's Test, the LRS Test for Distance Decline, the Radial Score Test, and Cuzick and Edwards' Test k = 7. For *N *= 500 and *N *= 1000 at *α*_2 _= 0.5 and 1, the Radial Score Test demonstrated the highest power among all focused cluster tests at lower values of *α*_1_. At these same *α*_1 _values, power for the Radial Score Test increased as *α*_2 _increased to 1. The Besag and Newell's Test at *k *= 4 and 7 for *N *= 500 and at *k *= 7 and 10 for *N *= 1000 showed a trend similar to the Radial Score Test but with consistently lower power. Tango's Test and the LRS Test for Distance Decline showed the highest power, though still poor, at higher *α*_1 _values. The power for these tests declined as *α*_2 _increased to 1. None of the other focused cluster tests we examined performed as well.

#### Data simulated with a directional effect (DIR) in relative risk

**Figure 3:**
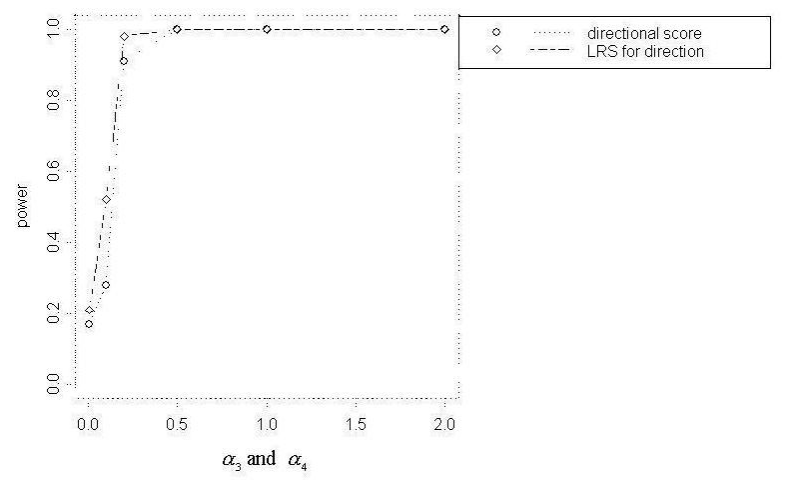
Power curves for DIR with *N *= 1000

The LRS Test for Direction demonstrated the highest power among the focused cluster tests we examined for detecting DIR clusters (Figure 3). The Directional Score Test showed similar power curves, reaching 100% power for each total event scenario. Besag and Newell's Test achieved modest power 30%, 40% and 40% respectively for *N *= 200 with *k *= 4, *N *= 500 with *k *= 10 and *N *= 1000 with *k *= 2. However other tests had less power. Generally, the tests showed less power at wider angles (*α*_3 _and *α*_4 _< 0.2) and increasing power as the angle narrowed.

#### Data simulated with a distance decline and directional effect (DDIR) in relative risk

**Figure 4:**
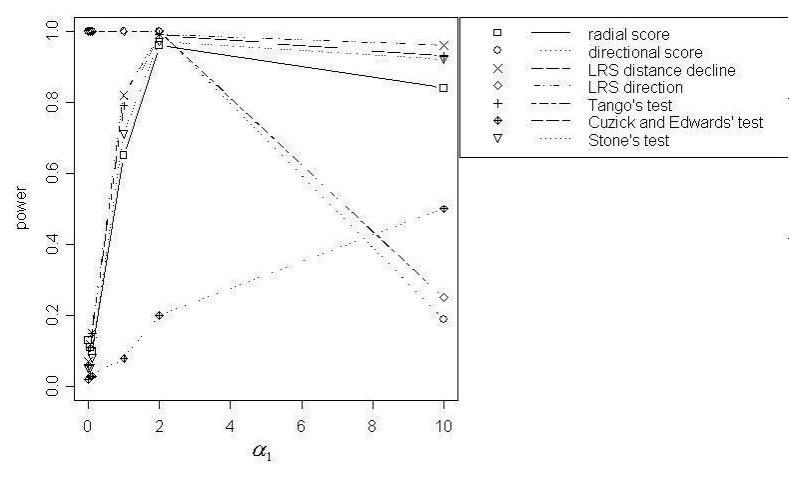
Power curves for DDIR, *α*_3 _and *α*_4 _= 0.5, with *N *= 1000

Figure 4 demonstrates the power curves for *N *= 1000 at *α*_3 _and *α*_4 _= 0.5. Power curves at the lowest *α*_3 _and *α*_4 _values were generally similar to the DD power curves, and power curves at the highest *α*_3 _and *α*_4 _values resembled the DIR power curves. Overall, the LRS Test for Direction was the best-performing test with respect to power at *α*_1 _< 1, and the Directional Score Test had similar power curves as *α*_3 _and *α*_4 _increased. Power for the LRS Test for Direction and for the Directional Score Test improved with increasing values of *α*_3 _and *α*_4_. Both tests attained 100% power for lower *α*_1 _values at *α*_3 _and *α*_4 _= 1 for 200 events and at *α*_3 _and *α*_4 _= 0.5 for *N *= 500 and *N *= 1000. For *α*_1 _≥ 1, Tango's Test and the LRS Test for Distance Decline, followed by the Radial Score Test and Stone's Test, showed the most power at lower *α*_3 _and *α*_4 _values. As *α*_3 _and *α*_4 _increased, these tests gradually increased in power, with all of these tests showing 100% power at the highest values for *α*_1_, *α*_3 _and *α*_4_. For these higher *α*_1 _values, power also improved for the Directional Score Test and the LRS Test for Direction as *α*_3 _and *α*_4 _increased. In most cases of higher *α*_1 _values, the power for both of these directional tests matched or surpassed the power for Tango's Test, the LRS Test for Distance Decline, the Radial Score Test and Stone's Test at the highest values of *α*_3 _and *α*_4_. Cuzick and Edwards' Test *k *= 7 and 10 also reached 100% power at the highest values for *α*_1_, *α*_3 _and *α*_4 _for *N *= 500 and *N *= 1000.

#### Data simulated with a peaked distance decline and directional effect (PDDIR) in relative risk

**Figure 5:**
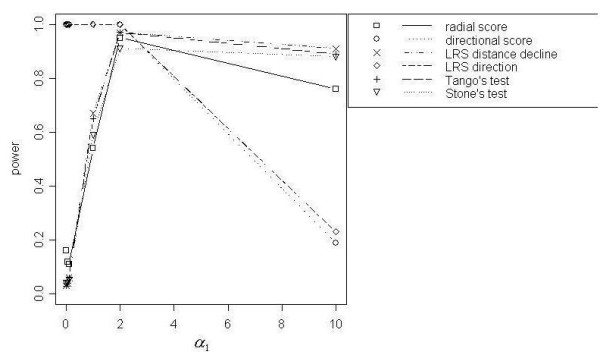
Power curves for PDDIR *α*_2 _= 0.05, *α*_3 _and *α*_4 _= 0.5, with *N *= 1000

We begin by presenting the findings for lower values of *α*_2 _(*α*_2 _= 0.05 and 0.1). Results for these two *α*_2 _levels were similar to one another and are represented by Figure 5. For *N *= 200, *N *= 500 and *N *= 1000, the best-performing tests for lower values of *α*_1 _(*α*_1 _< 1) were the LRS Test for Direction followed by the Directional Score Test. Though at lower *α*_3 _and *α*_4 _values, the highest power achieved was poor. As *α*_3 _and *α*_4 _increased, the LRS Test for Direction and the Directional Score Test improved in power. Tango's Test and the LRS Test for Distance Decline, followed by the Radial Score Test and Stone's Test, demonstrated the most power at higher levels of *α*_1 _(*α*_1 _> 0.1), increasing as *α*_3 _and *α*_4 _increased. However, at *α*_3 _and *α*_4 _= 0.5 and above, the LRS Test for Direction and the Directional Score Test generally demonstrated the highest power for all values of *α*_1 _except *α*_1 _= 10. At this *α*_1 _value, Tango's Test, the Radial Score Test, Stone's Test and the LRS Test for Distance Decline were more powerful until *α*_3 _and *α*_4 _reached 2; at which point, other tests matched their power. For *N *= 500, Cuzick and Edwards' Test at *k *= 7 and *k *= 10 also reached 100% power at *α*_3 _and *α*_4 _= 2 and *α*_1 _= 10. Power was low for all other values of these coefficients for this focused cluster test.

**Figure 6:**
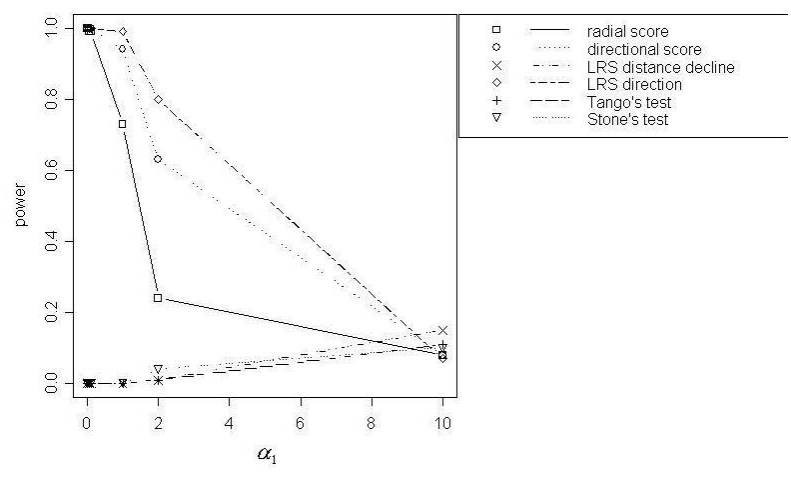
Power curves for PDDIR, *α*_2 _= 1, *α*_3 _and *α*_4 _= 0.5, with *N *= 1000

Figure 6 represents the power curves for higher values of *α*_2 _(*α*_2 _= 0.5 and 1), though results differed somewhat among the three total events scenarios. For *N *= 200, the LRS Test for Direction showed the best power overall. All focused cluster tests examined showed lower power for *α*_3 _and *α*_4 _= 0.05. But several tests improved as *α*_3 _and *α*_4 _increased to *α*_1 _= 2. Besag and Newell's Test at *k *= 7 and 10 showed similar power to the LRS Test for Direction at lower values of *α*_1_, *α*_3 _and *α*_4_. The Directional Score Test showed the second best power curve for values of *α*_3 _and *α*_4 _> 0.2. For each value of *α*_3 _and *α*_4_, the Directional Score Test, the LRS Test for Direction, and Besag and Newell's Test declined in power as values of *α*_1 _decreased. The Radial Score Test demonstrated an unusual U-shaped curve, most pronounced at *α*_3 _and *α*_4 _= 2. For *N *= 500 and *N *= 1000, the Radial Score Test showed the best power curve at *α*_3 _and *α*_4 _< 0.5, with power decreasing as *α*_1 _values declined. As *α*_3 _and *α*_4 _increased from 0.5, the LRS Test for Direction demonstrated the highest power, followed by the directional score and Radial Score Tests. For each of these tests, power decreased as *α*_1 _increased. Tango's Test and the LRS Test for Distance Decline showed an unusual trend of no power at values of *α*_1 _under 10, then a sudden increase in power at *α*_1 _= 10. This trend became more pronounced as *α*_3 _and *α*_4 _increased.

### Focused cluster model results

**Figure 7:**
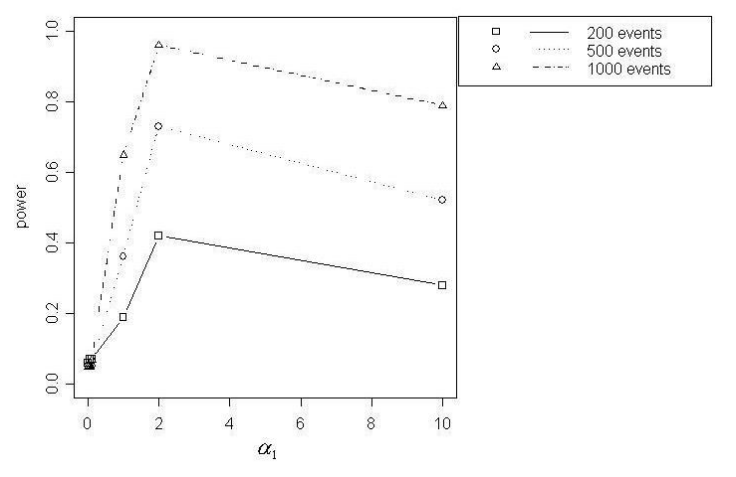
Null model vs. DD model

**Figure 8:**
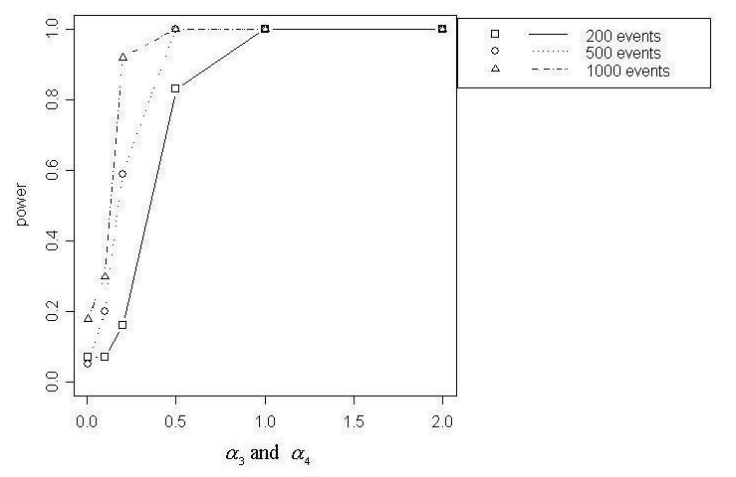
Null model vs. DIR model

**Figure 9:**
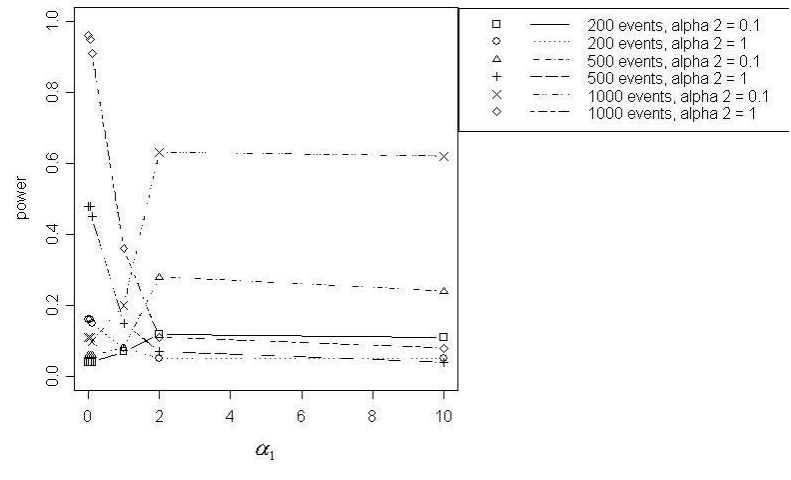
Null model vs. PDD model

**Figure 10:**
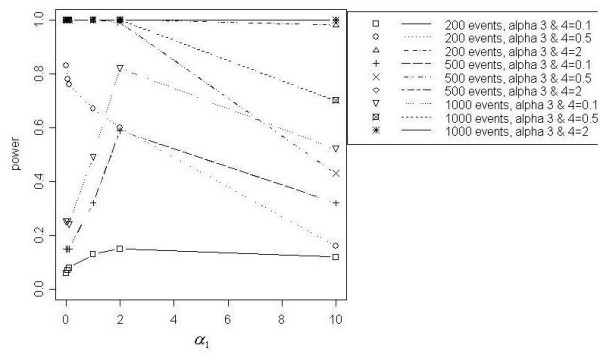
Null model vs. DDIR model

Figures 7 through 11 demonstrate that better power curves were achieved for larger numbers of events. Figure 7 shows the power curve resulting from comparing the alternative hypothesis model that included a function for a distance decline in relative risk to the null hypothesis model with null relative risk. Power was low when the *α *coefficients were small, peaked when *α*_1 _= 2 and decreased slightly as *α*_1 _increased to 10. For 200 events, low to moderate power was achieved; however, better power was evident for 500 and 1000 events. Use of the null model versus the model with a function of directional elevated relative risk showed power increasing with increasing values of *α*_3 _and *α*_4_, or as the angle of effect narrowed (Figure 8). One hundred percent power was achieved in each of the three total event situations (N = 200, 500 and 1000). Figure 9 shows that power was inversely related to the *α*_1 _coefficient value when *α*_2 _was larger, or the peak was further from the source (*α*_2 _= 1). Power increased directly with *α*_1 _values when *α*_2 _was smaller, or the peak was closer to the source (*α*_2 _= 0.1). Only the 1000 event situation achieved moderate to high power. Overall, better power curves were evident for the comparisons between null models and the models that included a function for distance decline and directional relative risk (Figure 10). For all variations of total events, 100% power was achieved across all *α*_1 _values for higher values of *α*_3 _and *α*_4 _(*α*_3 _and *α*_4 _= 2). At *α*_3 _and *α*_4 _= 0.5, 100% power was shown for the 500 and 1000 event situations at lower *α*_1 _values (*α*_1 _< 10), however power decreased at the highest value of *α*_1 _(*α*_1 _= 10) for 500 and 1000 events. For 200 events, lower overall power was achieved, and power began decreasing immediately with increasing *α*_1 _values. At the lowest *α*_3 _and *α*_4 _values (*α*_3 _and *α*_4 _< 0.5), power increased directly with values of *α*_1 _but decreased at *α*_1 _= 10 for 500 and 1000 events. Results from the final null model comparison, the null model versus a model including a spatial function for peaked distance decline and direction, is shown in Figure 5. For 1000 events and *α*_3 _and *α*_4 _= 2, the power of the test from the alternative model was more powerful; and the 500 event situation was similar, with power decreasing slightly at *α*_1 _= 10. The overall trend for all three total event counts at *α*_3 _and *α*_4 _= 0.5 showed very good power at lower levels of *α*_1 _(*α*_1 _< 10 for 500 and 1000 events, *α*_1 _< 1 for 200 events), but power decreased with increasing values of *α*_1_. At *α*_3 _and *α*_4 _= 0.1a different overall trend was evident, with power generally increasing directly with values of *α*_1_. Power trends for *α*_2 _= 1, for all *α*_3 _and *α*_4 _values and all total event counts, demonstrated an inverse relationship between power and *α*_1 _value. Additionally, power increased with increasing values of *α*_3 _and *α*_4_.

**Figure 11:**
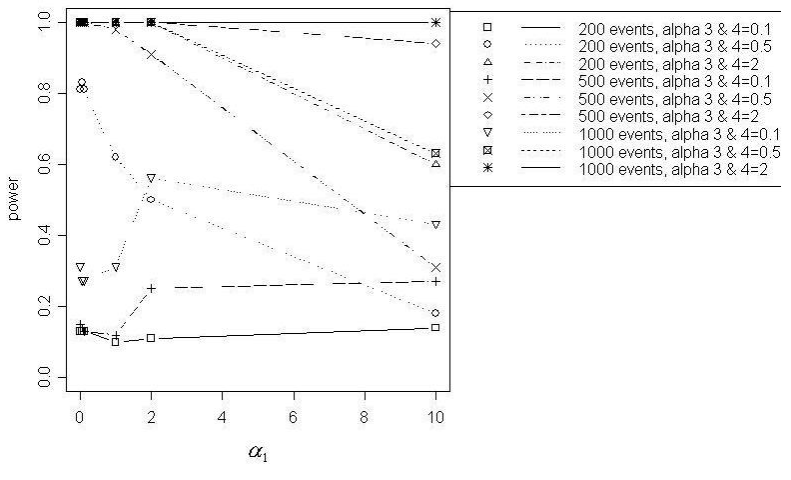
Null model vs. PDDIR model, *α*_2 _= 0.1

Comparison of the distance decline relative risk model to a model including a peaked distance decline function showed that no power was gained from adding the parameter for a peak. Power was less than 10% for all total event scenarios and variations of the *α *coefficients. However, better power curves resulted from other model comparisons. At values of *α*_3 _and *α*_4 _= 0.5 and 2, adding direction as a parameter to models with a function for distance decline vastly improved power for detecting clusters with distance decline and directional distributions. The increase in power was low for the addition of the directional parameter when *α*_3 _and *α*_4 _= 0.1. Similar trends were evident for the addition of parameters for a peak and for direction when compared to a distance decline only relative risk model. In general, power was high at all but the highest *α*_1 _value (*α*_1 _= 10) when *α*_3 _and *α*_4 _= 0.5 and 2. Yet, improvements in power were not very large for lower levels of *α*_3 _and *α*_4 _(*α*_3 _and *α*_4 _< 0.2).

Under certain conditions, power greatly increased when parameters were added to a directional only model. When comparing a directional only model to a model with parameters for distance decline and direction, power tended to increase with increasing *α*_1_, *α*_3 _and *α*_4 _values. However a slight decrease in power was evident at *α*_1 _= 10 for most model comparisons. The power curves were very similar to those for the comparison between a directional-only model to models with parameters for peaked distance decline and direction at lower values of *α*_2 _(*α*_2 _< 0.5). At higher levels of *α*_2 _(*α*_2 _> 0.1), power trends differed greatly, with the highest power at lowest levels of *α*_1 _and greater decreases as *α*_1 _increased to 10.

Power curves from models with parameters for peaked distance decline were also compared to models with parameters for peaked distance decline and direction. Generally, power trends were similar for the two *α*_2 _levels with power inversely related to *α*_1 _value and directly related to *α*_3 _and *α*_4 _values. The improvement in power was very low for the addition of the directional component at the lowest *α*_3 _and *α*_4 _values (*α*_3 _and *α*_4 _< 0.2).

As with previous model comparisons, the addition of a parameter for peak generally resulted in low power when parameters for distance decline and direction were already in the model. The highest power achieved was modest (about 40%) and was evident only for 1000 events at the highest values for *α*_2_, *α*_3 _and *α*_4 _values (*α*_2 _= 1, *α*_3 _and *α*_4 _= 2) at *α*_1 _= 1.

## Conclusion

### Focused cluster tests

Based on the results presented, we found that Tango's Test, the LRS Test for Distance Decline, Stone's Test and the Radial Score Test showed the most power for detecting a significant difference in relative risk simulated with DD from a centrally-located pollution source at a fixed location. As expected, power increased directly with number of total cases of disease involved in the cluster. For these tests, power also tended to increase as the slope of the DD became steeper or, in other words, as the relative risk changed more rapidly with increasing distance from the pollution source. The results of our power evaluations are not surprising as the best-performing cluster tests for detecting radial clusters with DD were generally developed for detecting these cluster shapes.

Power for detecting PDD clusters with *N *= 200 was generally low, with reasonable power demonstrated at *N *= 500 and *N *= 1000 for some combinations of peak distances from the pollution source and slopes of decline in risk. Evaluating the power of focused cluster tests to detect PDD clustering with peaks closest to the source revealed that the LRS Test for Distance Decline, Tango's Test, Stone's Test and the Radial Score Test showed the highest power among the tests evaluated. Power generally increased for these tests as the slope of the cluster became steeper. This trend reversed as peaks increased in distance from the pollution source so that power generally decreased as the slope became steeper. The Radial Score Test demonstrated the most power for detecting peaks furthest from the pollution source for *N *= 500 and *N *= 1000, while Besag and Newell's Test performed best for *N *= 200.

Supporting our hypothesis that focused cluster tests containing functions appropriate to the spatial pattern of pollution dispersion would be more powerful, the LRS test for direction and the Directional Score Tests revealed the most power for detecting focused clusters with DIR. Power increased for these tests with increased number of total events and as the angle of effect became more pronounced.

For data simulated with DDIR, the LRS Test for Direction performed best with wider angles of effect and the flattest declines in slope. But as the angle of effect became stronger, the LRS Test for Direction was powerful at all slopes. Overall, power to detect clusters with a DDIR distribution generally increased with narrower angles of effect and as the total number of events simulated increased. As the slope of the decline became steeper, Tango's Test, LRS Test for Distance Decline, Stone's Test and the Radial Score Test showed comparable power. Power for the Directional Score Test greatly improved with stronger angles of effect, becoming one of the best-performing tests for all slope levels. To summarize the power evaluation results for detecting DDIR clusters, directional tests demonstrated the most power with flatter declines in cluster slopes; whereas the radial distance decline tests were more powerful with steeper declines in slope. Both types of test improved with narrowing angles of effect. With those cluster patterns, directional tests became more powerful at all slope levels and distance decline tests remained powerful with steeper slopes.

In order to simulate data with clusters of PDDIR, only one component was added to the simulation equation for clusters of DDIR: *α*_2 _* log(*d*). Therefore, as one would expect, at very low values of *α*_2 _(e.g. 0.005), the results of power evaluations for detecting PDDIR clusters were very similar to those for detecting DDIR clusters. However, as *α*_2 _values increased, or as the peak of the cluster increased in distance from the pollution source, findings differed. With the widest angles, overall power for detecting PDDIR with *N *= 200 was low. At these same angles, the Radial Score Test proved best for detecting clustering with *N *= 500 and *N *= 1000. This test decreased in power with increasing steepness of slope. As the angle of the directional effect narrowed, the LRS Test for Direction and the Directional Score Test also showed comparable power, particularly with a greater number of events. The power for these tests decreased as the cluster slope flattened. Interestingly, the Radial Score Test also followed this trend and demonstrated power second only to the directional tests. These results show that although the Radial Score Test could detect DIR effects combined with PDD effects, it was generally less powerful than directional tests at the narrowest angles of effect.

### Focused cluster models

Overall, the addition of model parameters for peaks did not appear to contribute to improved power, particularly when a distance decline parameter was already included in the model. Only at *α*_1 _< 1, or the flattest slope, did the null versus peaked distance decline model comparison show higher power than the null versus distance decline model comparison. Also, very low power resulted when comparing the distance decline only model to the peaked distance decline model and when adding a parameter for peak to models already containing distance decline and directional parameters. Lastly, the power curves in which only a directional component was added are very similar to those resulting from the addition of peaked and directional components. Low power to detect a peaked distance decline cluster of elevated risk may be related to the variation in relative risk. For example, Figure 3 shows higher power for lower values of *α*_1 _when *α*_2 _= 1. For *α*_2 _= 1, the range of relative risk is much greater at lower values of *α*_1 _and decreased directly with *α*_1_.

The addition of a directional parameter improved the power of tests from models, particularly for detecting disease clusters distributed over narrower angles of effect. Evidence of this outcome was provided by the power curves resulting from the comparison between the model of null relative risk and the model with a directional component. When a directional parameter was added to distance decline models and to peaked distance decline models, power was very high for clustering with gentler declines (*α*_1 _< 2) and narrower angles (*α*_3 _and *α*_4 _> 0.1). However, power tended to decrease as *α*_1 _increased from 2 to 10 and as the angle of effect widened, or as *α*_3 _and *α*_4 _increased.

Similarly, distance decline also appears to be an important parameter to include in focused cluster models, with respect to power. In opposition to the directional component, parameters for distance decline appeared most beneficial with clusters of steeper declines (larger *α*_1 _values). However, this observation may be due to the main effect extending outside the window of the simulated region for clusters with gentler declines. Comparison of the null model to the distance decline model showed power generally increasing as the steepness of the cluster slope increased. Also, the addition of a parameter for distance decline to a directional model showed similar power curves. One interesting effect in many of the model comparisons involving a distance decline parameter was the decrease in power from *α*_1 _= 2 to *α*_1 _= 10. This may be caused by the steepness of the slope resulting in fewer data points demarcating the decline in slope. As the number of observations decreases, power will also decrease.

### Overall conclusions

Though a variety of spatial scales and shapes of clusters were examined, further power evaluations are needed in order to explore fully the range of disease clustering that could result from various pollution dispersion spatial patterns. Given the overall finding that more complex spatial cluster patterns can be more difficult to detect, the development and use of additional sampling schemes for power evaluations of these clusters would be beneficial. We chose to simulate three total numbers of events because count data are typically most accessible in cluster investigations. Work is currently underway to examine power for case-event data, however further investigations are needed to examine a greater range of total numbers of events for count data. Smaller numbers of total events are of particular importance in cluster investigations of rare diseases or sparsely populated areas. A number of variations were examined for the four *α *coefficients, yet a more comprehensive range of coefficients, representing additional changes in shape and scale, would provide important information. Other spatial components also should be examined, such as azimuth, which could be of primary concern for air pollution sources located in valleys. Additionally, these power evaluations were performed simulating dispersion from a single, centrally-located pollution source. Further power evaluations are needed to address cluster detection in situations where pollutants are dispersed from multiple sources. Williams and Ogston [8] compared observed and simulated spatial distributions of environmental exposures. Additional comparisons between measured levels of environmental pollutant spatial dispersion with those simulated here would also be useful in evaluating the accuracy of spatial functions in a variety of situations. Similar power evaluations of focused cluster tests and models using individual-level data are also needed to improve cluster investigation techniques, and work in this area is proceeding.

It should also be noted that the results from this investigation are subject to Monte Carlo error; though this error has a maximum value of 0.05 with a sample size of 100 simulated data sets. In order to estimate the uncertainty, we determined the Monte Carlo standard errors for the power estimates. Table 2 provides examples of these error estimates for the focused cluster tests, and Table 3 demonstrates the errors for the tests from the fitted models. If an approximate normal distribution is assumed for the power estimates, confidence intervals could be computed via standard procedures. However, the overall conclusions of this investigation were based on many results and should not be overly influenced by the level of error.

**Table 2:** Sample Monte Carlo standard errors for focused cluster tests with N = 1000

	**Radial Score Test**	**DIR Score Test**	**Besag and Newell's Test (*k *= 7)**	**Cuzick and Edwards' Test (*k *= 7)**	**Tango's Test (*τ *= 5)**	**LRS DIR Test**	**LRS DD Test**	**Stone's Test**
**DD**								
*α*_1 _= 0.005	0.02	0.02	0.01	0.02	0.02	0.02	0.02	0.02
*α*_1 _= 0.05	0.02	0.02	0.01	0.02	0.03	0.02	0.03	0.02
*α*_1 _= 0.1	0.02	0.02	0.01	0.02	0.03	0.02	0.03	0.03
*α*_1 _= 1	0.05	0.02	0.01	0.03	0.04	0.02	0.04	0.05
*α*_1 _= 2	0.03	0.02	0.01	0.04	0.02	0.03	0.01	0.03
*α*_1 _= 10	0.04	0.02	0.01	0.05	0.03	0.02	0.03	0.03

**DIR**								
*α*_3 _and *α*_4 _= 0.005	0.03	0.04	0.01	0.02	0.03	0.04	0.03	0.02
*α*_3 _and *α*_4 _= 0.1	0.03	0.04	0.02	0.02	0.02	0.05	0.02	0.02
*α*_3 _and *α*_4 _= 0.2	0.03	0.03	0.01	0.01	0.03	0.01	0.03	0.02
*α*_3 _and *α*_4 _= 0.5	0.03	0.00	0.02	0.01	0.02	0.00	0.02	0.02
*α*_3 _and *α*_4 _= 1	0.03	0.00	0.03	0.00	0.03	0.00	0.03	0.02
*α*_3 _and *α*_4 _= 2	0.03	0.00	0.04	0.00	0.04	0.00	0.04	0.03

**PKDD ***α*_1 _= 0.005								
*α*_2 _= 0.05	0.03	0.02	0.02	0.02	0.01	0.02	0.02	0.01
*α*_2 _= 0.1	0.04	0.02	0.02	0.01	0.01	0.02	0.01	0.00
*α*_2 _= 0.5	0.03	0.02	0.04	0.01	0.00	0.02	0.00	0.00
*α*_2 _= 1	0.01	0.02	0.04	0.01	0.00	0.02	0.00	0.00

**Table 3:** Sample Monte Carlo standard errors for fitted models with N = 1000

**Null vs. DD**	*α*_1 _= 0.005	*α*_1 _= 0.05	*α*_1 _= 0.1	*α*_1 _= 1	*α*_1 _= 2	*α*_1 _= 10
Standard Error	0.02	0.02	0.03	0.05	0.02	0.04

**Null vs. DIR**	*α *_3 _and *α*_4 _= 0.005	*α*_3 _and *α*_4 _= 0.1	*α*_3 _and *α*_4 _= 0.2	*α*_3 _and *α*_4 _= 0.5	*α*_3 _and *α*_4 _= 1	*α*_3 _and *α*_4 _= 2
Standard Error	0.04	0.05	0.03	0.00	0.00	0.00

**Null vs. PKDD ***α*_1 _= 0.005	*α*_2 _= 0.05	*α*_2 _= 0.1	*α*_2 _= 0.5	*α*_2 _= 1		
Standard Error	0.03	0.03	0.04	0.00		

This study examined the power of a number of focused cluster tests and generalized linear models to detect a wide range of simulated focused cluster shapes and scales. The results of this study provide information that can improve the choice of statistical method in focused cluster investigations. To summarize the overall findings from this investigation:

1) Focused cluster tests and tests from models containing functions appropriate to the spatial pattern of pollution dispersion are more powerful. DIR tests were more powerful detecting clusters with narrower angles of effect and DD tests were more powerful detecting clusters with steeper declines in slope.

2) Power increased with stronger DD (steeper slopes) and DIR (narrower angles) effects.

3) Power for detecting clusters with peaked effect patterns was generally low.

## Methods

### Data simulation

As count data are generally more widely available in disease mapping studies than individual-level data, count data were simulated for this study. A basic Poisson model was assumed for the counts: *y*_*i *_~ Poisson(*e*_*i *_*θ*_*i*_), where an expected count *e*_*i *_is modified by a relative risk *θ*_*i *_and *y*_*i*_, *i *= 1,...,*M*, is the count of disease in the *i*th region. Clusters of three sizes (*N *= 200, 500 and 1000 events) were simulated from the multinomial distribution, where the probability of a case in the *i*th region is .

Five shapes of clusters were simulated, including: 1) distance decline, where risk declines with increasing radial distance from the pollution source (DD, Model 1, Additional File 1: Simulated models of the five focused cluster shapes); 2) peaked distance decline, where risk peaks and then declines with increasing radial distance from the source (PDD, Model 2, Additional File 1); 3) direction, increasing disease risk in a particular angular direction from the pollution source (DIR, Model 3, Additional File 1); 4) distance decline combined with a directional effect (DDIR, Model 4, Additional File 1); and 5) peaked distance decline combined with a directional effect (PDDIR, Model 5, Additional File 1). These shapes correspond to frequently encountered air pollution dispersion patterns from point sources. For instance, shape 1 typifies dispersion from a ground level source with a relatively uniform distribution of wind directions, shape 3 represents ground level dispersion with a dominant wind direction and shape 5 represents dispersion from an elevated source with a dominant wind direction.

The count data simulated for these five cluster shapes under the alternative hypothesis as well as data simulated under the null hypothesis of randomly distributed counts of disease were assigned to regional centroids of a 16*16 unit square grid. The grid of 256 regions of uniform size and shape, unitless in geographic terms, also contained a centrally located pollution source. Expected disease rates were considered to be uniform throughout the regions composing the simulated study area. The model for the relative risk at location *x*, *θ*(*x*, *β*) represents the relationship between the pollution source and spatial distribution of associated disease, for some choice of parameters *β*. As shown in Additional File 1: Simulated models of the five focused cluster shapes, coefficients in the model equations were varied in order to represent further variations of scale for the five main cluster shapes (DD, PDD, DIR, DDIR, and PDDIR).

### Power evaluation methods for focused cluster tests

In this study, we evaluated the power to detect the five general cluster shapes for eight widely known focused cluster tests, including: Stone's Maximum Likelihood Test [4], the focused adaptation of Besag and Newell's Test [1], Cuzick and Edwards' Test [9], Tango's Focused Test [5], variations of the Lawson-Waller Score Test [7,10], and variations of Bithell's Linear Risk Score (LRS) Test [11]. Power was evaluated through the use of Monte Carlo significance testing with 100 datasets simulated under the null hypothesis and 100 datasets simulated under each alternative hypothesis (i.e., each variation of cluster shape, size and scale). The formulations used for each test are briefly described.

For Stone's Maximum Likelihood Test [4] (hereafter referred to as Stone's Test), we selected a number of distances (*d*_1_,...,*d*_*k*_) as bins and placed regions falling between these distances in the appropriate bin. Stone's Test was then defined as follows:



where  is the vector of maximum likelihood estimates under the alternative hypothesis of decreasing risk with increasing distance from the cluster center, and *θ*_0 _is the relative risk under the null hypothesis of constant risk.

For the purposes of this investigation, the focused cluster adaptation of Besag and Newell's Test [1], Waller and Lawson reference} was defined as: *M *= min(*i *: *D*_*i *_≥ *k*), where *D*_*i *_is the number of cases accumulated among *i *regions and *k *is defined as the number of cases specified to define a cluster. Four variations of *k *(2, 4, 7 and 10) were evaluated.

In the one sample approach of Cuzick and Edwards' Test [9], data are ordered by distance to cluster center; and the test statistic is defined as:



where *n*_0 _is the number of regions required until we have *k *events (*k *= the number of cases designating a cluster). We examined four values of *k*: 2, 4, 7 and 10. The application of this test involves the construction of increasingly larger circles around the point source of interest until the number of cases in the regions contained by the circle equals *k *cases.

We applied the following formulation of Tango's Focused Test [5]:

*C*_*F *_= *A*(*r *- *p*),

where, *A *is a vector with *i*th element given by *a*_*i *_= exp(-*d*_*i*_/*τ*), *d*_*i *_= distance of the i th region centroid from the pollution source, and *r *and *p *are vectors with *i*th element *y*_*i *_/ *N *and *e*_*i *_/ *N *respectively. For the purposes of this study, *τ *was defined as 1 and 5.

Bithell [11] indicates that LRS Tests can incorporate various functions of distance and rank to describe exposure. We applied functions of distance decline and direction, as described by Lawson [7], to represent exposure to environmental contaminants from a centrally located pollution source. Bithell's LRS Test statistic formulation [11] is described as:



where *θ*_1*i *_is the area-specific relative risk based on the alternative hypothesis. We therefore defined:

*H*_1_: distance decline in risk and:  for *H*_1_: directional effect of risk where *μ *= the mean angle between the regional centroid and the pollution source.

Two formulations of the Lawson-Waller Score Test [7,10] also were evaluated. We applied the Radial Distance Decline Score Test as defined by Lawson [7]:



Lawson's [7] formulation of the Directional Score test also was used:



where *μ *is the mean angle estimated under the null hypothesis during model fitting; however, during data simulation,  was selected.

### Power evaluation methods for focused cluster tests from models

Power evaluations of tests from generalized linear Poisson regression models to detect focused clustering were conducted with Monte Carlo significance testing of differences in model residual deviances. These regression models take the form: *θ *(*x*_*i*_, *β*) = exp(*x*_*i*_' *β*) where *x*_*i *_is the region centroid, *x*_*i*_' is the *i *th row of a covariate design matrix (that can include *x*_*i*_), and *β *is a parameter vector describing the relationship between the spatial distribution of disease and the pollution source. Detailed equations of the fitted models are presented in Additional File 2: Fitted models for the five focused cluster shapes. For Monte Carlo testing, models with fewer parameters describing the spatial cluster shape were compared to models containing more parameters, or a more comprehensive function describing the spatial cluster pattern. Essentially, the resulting power curves represent the capability of detecting disease clusters when more complete information regarding the cluster pattern shape and scale is included in the model. A list of the model comparisons is presented in Table 1. For each of these power evaluations, the model with fewer spatial parameters was fit, and any *α *coefficients were estimated. The comparison model, containing additional spatial parameters to describe the spatial cluster pattern, was then fitted, including fixed *α *estimates obtained from fitting the model with fewer spatial terms. The residual deviance difference between the two models was then determined. In order to obtain the critical value for the Monte Carlo testing, the previously described procedure was followed using 100 datasets simulated with no disease clustering to represent the expected counts and 100 datasets simulated under the model with fewer spatial parameters to represent the observed counts. The difference of the residual deviances obtained from comparing the two models was determined as the critical value. This procedure was repeated using the 100 datasets simulated with no disease clustering to represent the expected counts and the 100 datasets simulated under the alternative hypothesis of the more explicit spatial distribution to represent the observed counts. The 100 residual deviance differences were then compared to the critical value obtained from the null hypothesis testing. The number of test statistics under the alternative hypothesis that were greater than the critical value provided the power estimate.

**Table 1:** Model comparisons

**Base Model**	**Spatial Distribution Function of Alternative Model**	**Base Model Observed Dataset**	**More Explicit Model Observed Dataset**
Null	Distance Decline	Relative Risk = 1	Distance Decline Relative Risk
Null	Direction	Relative Risk = 1	Directional Relative Risk
Null	Peaked Distance Decline	Relative Risk = 1	Peaked Distance Decline Relative Risk
Null	Distance Decline and Direction	Relative Risk = 1	Distance Decline and Direction Relative Risk
Null	Peaked Distance Decline and Direction	Relative Risk = 1	Peaked Distance Decline and Direction Relative Risk
Distance Decline	Distance Decline and Direction	Distance Decline Relative Risk	Distance Decline and Direction Relative Risk
Distance Decline	Peaked Distance Decline	Distance Decline Relative Risk	Distance Decline and Direction Relative Risk
Distance Decline	Peaked Distance Decline and Direction	Distance Decline Relative Risk	Peaked Distance Decline and Direction Relative Risk
Direction	Distance Decline and Direction	Directional Relative Risk	Distance Decline and Direction Relative Risk
Direction	Peaked Distance Decline and Direction	Directional Relative Risk	Peaked Distance Decline and Direction Relative Risk
Peaked Distance Decline	Peaked Distance Decline and Direction	Peaked Distance Decline Relative Risk	Peaked Distance Decline and Direction Relative Risk
Distance Decline and Direction	Peaked Distance Decline and Direction	Distance Decline and Direction Relative Risk	Peaked Distance Decline and Direction Relative Risk

## Authors' contributions

RCP carried out the simulations, performed the statistical analysis and drafted the manuscript. ABL conceived of the study and acquired funding. ABC assisted with programming the simulations and the statistical analyses. DEP, TEA, CEF, ABC, ABL and JRH had scientific input from the project's inception, through early development of the protocol, and in drafting the manuscript. All authors read and approved the version of the manuscript submitted here.

## Supplementary Material

Additional File 1 Simulationmodels.pdf contains simulation models of the five focused clusters shapes Click here for file

Additional File 2 Fittedmodels.pdf contains fitted models for the five focused cluster shapes Click here for file
