# Supplementary material for: Scale and shape issues in focused cluster power for count data
Source: Int J Health Geogr. 2005 Mar 31;4:8. doi: 10.1186/1476-072X-4-8 (PMC1079923; doi:10.1186/1476-072X-4-8)
Supplement: Additional File 1 — Simulationmodels.pdf contains simulation models of the five focused clusters shapes [file 1476-072X-4-8-S1.pdf]

1) DD, where: $\theta = 0.5[1 + \exp(-\alpha_1 d)]$

α_1 = 0.005, 0.05, 0.1, 1, 2, 10

2) PDD, where: $\theta = 0.5[1 + \exp\{(\alpha_2 \log(d)) - (\alpha_1 d)\}]$

α_1 = 0.005, 0.05, 0.1, 1, 2, 10

α_2 = 0.05, 0.1, 0.5, 1

3) DIR, where: $\theta = 0.5\left[1 + \exp\left\{\left(\alpha_3 \cos(\phi - \mu)\right) + \left(\alpha_4 \sin(\phi - \mu)\right)\right\}\right]$

where the pollution source is arbitrarily selected to emit a contaminant in the direction of the mean angle $\mu = \frac{\pi}{4}$ and where ϕ = the angle of the centroid from the pollution source,

α_3 = 0.05, 0.1, 0.2, 0.5, 1, 2

α_4 = 0.05, 0.1, 0.2, 0.5, 1, 2

4) DDIR, where: $\theta = 0.5\left[1 + \exp\left\{\left(\alpha_3 \cos(\phi - \mu)\right) + \left(\alpha_4 \sin(\phi - \mu)\right) - \left(\alpha_1 d\right)\right\}\right]$

α_1 = 0.005, 0.05, 0.1, 1, 2, 10

α_3 = 0.05, 0.1, 0.2, 0.5, 1, 2

α_4 = 0.05, 0.1, 0.2, 0.5, 1, 2

5) PDDIR, where:

$$\theta = 0.5\left[1 + \exp\left\{\left(\alpha_3 \cos(\phi - \mu)\right) + \left(\alpha_4 \sin(\phi - \mu)\right) + \left(\alpha_2 \log(d)\right) - \left(\alpha_1 d\right)\right\}\right]$$

α_1 = 0.005, 0.05, 0.1, 1, 2, 10

α_2 = 0.05, 0.1, 0.5, 1

α_3 = 0.05, 0.1, 0.2, 0.5, 1, 2

α_4 = 0.05, 0.1, 0.2, 0.5, 1, 2
