# Supplementary material for: Scale and shape issues in focused cluster power for count data
Source: Int J Health Geogr. 2005 Mar 31;4:8. doi: 10.1186/1476-072X-4-8 (PMC1079923; doi:10.1186/1476-072X-4-8)
Supplement: Additional File 2 — Fittedmodels.pdf contains fitted models for the five focused cluster shapes [file 1476-072X-4-8-S2.pdf]

1) DD, where: $\theta = 0.5[1 + \exp(-\alpha_1 d)]$

d =distance

2) PDD, where: $\theta = 0.5[1 + \exp\{(\alpha_2 \log(d)) - (\alpha_1 d)\}]$

3) DIR, where: $\theta = 0.5\left[1 + \exp\left\{\left(\alpha_3 \cos(\phi - \mu)\right) + \left(\alpha_4 \sin(\phi - \mu)\right)\right\}\right]$

ϕ = the angle of emission distribution from the pollution source to each regional centroid and μ is the estimated mean angle.

4) DDIR, where: $\theta = 0.5\left[1 + \exp\left\{\left(\alpha_3 \cos(\phi - \mu)\right) + \left(\alpha_4 \sin(\phi - \mu)\right) - \left(\alpha_1 d\right)\right\}\right]$

5) PDDIR, where:

$$\theta = 0.5\left[1 + \exp\left\{\left(\alpha_3 \cos(\phi - \mu)\right) + \left(\alpha_4 \sin(\phi - \mu)\right) + \left(\alpha_2 \log(d)\right) - \left(\alpha_1 d\right)\right\}\right]$$
